# Cascaded metasurface for polarization-dependent varifocal vortex beam manipulation

**DOI:** 10.1515/nanoph-2025-0153

**Published:** 2025-07-09

**Authors:** Wenhui Xu, Chenhui Zhao, Hui Li, Jie Li, Qi Tan, Yufei Liu, Hang Xu, Yun Shen, Jianquan Yao

**Affiliations:** Key Laboratory of Opto-Electronics Information Technology (Tianjin University), Ministry of Education, 12605 School of Precision Instruments and Opto-Electronics Engineering, Tianjin, 300072, China; Department of Physics, School of Physics and Materials Science, Nanchang University, Nan Chang, 330031, China; Sichuan Meteorological Optoelectronic Sensor Technology and Application Engineering Research Center, Chengdu University of Information Technology, Chengdu, 610225, China

**Keywords:** vortex beam, cascaded metasurface, polarization-multiplexing, focal adjustment

## Abstract

Vortex beams, characterized by orbital angular momentum (OAM), hold significant potential in optical communications, quantum information processing, and optical manipulation. However, existing metasurface designs are largely confined to single-degree-of-freedom control, such as static OAM generation or fixed focal points, which limiting their ability to integrate polarization multiplexing with dynamic focal tuning. To address this challenge, we propose a tunable multifunctional cascaded metasurface that synergizes polarization-sensitive phase engineering with interlayer rotational coupling, overcoming conventional device limitations. The designed metasurface independently generates distinct OAM states in orthogonal circular polarization channels under right-handed circularly polarized (RCP) incidence, that is, a vortex beam with topological charge ℓ = −1 in the left-handed circularly polarized (LCP) channel and a superimposed vortex state (ℓ = +1, −1) in the RCP channel. Continuous focal tuning is achieved via interlayer rotation in the axis-direction, with experimental validation at target frequency. Experimental results demonstrate the focal length modulation range from 25.9λ to 9.5λ as the interlayer rotation angle varies between 90° and 240°. This multi-degree-of-freedom control strategy establishes a new method for high-capacity optical communications, dynamic holography, and quantum state manipulation, while advancing the development of intelligent metasurfaces for 6G networks and integrated photonic systems.

## 1 Introduction

The terahertz (THz) regime, occupying the gap between microwave and infrared frequencies, has become indispensable for advanced applications including high-speed wireless communication [1], [2], [3], nondestructive sensing [4], [5], [6], [7], and multidimensional imaging [8], [9], [10]. Despite this potential, precise dynamic manipulation of THz wavefronts, particularly concurrent regulation of focal characteristics and OAM states, remains fundamentally constrained by the limited adaptability of traditional diffractive elements. Metasurface, composed of subwavelength meta-atoms to locally engineer electromagnetic properties, has revolutionized wavefront shaping through spatially tailored phase discontinuities [11], [12], [13], [14], [15], [16], [17], [18], [19]. However, most THz metasurfaces predominantly exhibit static operational modes, restricted focal adaptability, and insufficient fusion of multiple optical degrees of freedom such as polarization diversity, axial focusing, and OAM multiplexing [20], [21], [22].

Existing reconfigurable metasurface platforms utilizing electrical, thermal, or chemical mechanisms present inherent operational limitations. Voltage-driven configurations employing liquid crystals or phase-change materials, though capable of millisecond-scale switching, typically demand high drive potentials and suffer from chromatic aberrations across multioctave THz bands [23], [24]. Thermally tuned systems, while spectrally broadband, are plagued by slow thermal relaxation and irreversible hysteresis effects [25], [26], [27]. The method of chemical regulation faces significant challenges, as the metamaterials undergo continuous chemical transformations that can lead to structural degradation and other related issues. The longevity and response time of this approach currently stand out as the primary concerns requiring urgent attention [28], [29], [30]. Crucially, these approaches lack independent control channels for polarization state manipulation during focal parameter tuning. Cascaded metasurfaces, leveraging relative rotational alignment between stacked layers, have emerged as a mechanically reconfigurable platform for focal tuning [31], [32], [33]. By rotating constituent layers, the Moiré effect generates spatially varying phase gradients, enabling dynamic focal shifts without requiring external power sources or complex control systems. However, conventional cascade designs provide the basis for metasurface optimization. However, Moiré phase modulation does not enable independent control of different spin polarization channels, limiting the metasurface’s freedom to modulate THz multidimensional optical fields [34], [35], [36], [37].

In this work, we propose a cascaded metasurface that synergistically combines polarization-selective phase manipulation and rotational focal tuning to achieve multifunctional THz wavefront control. The device comprises two distinct layers. The polarization-selective Layer I featuring rectangular meta-atoms optimized for spin-decoupled phase modulation. This layer enables independent phase profiles for RCP and LCP channels, while introducing spiral phase gradients for OAM generation. Another layer is a polarization-insensitive Layer II with rotationally tunable cylindrical meta-atoms. Mechanical rotation of this layer (90°–240°) dynamically modulates the effective focal length. The interlayer relative rotation facilitates continuous focal shift from 25.9λ to 9.5λ (experimentally), which also enables the generation of topological charges ℓ = −1 in the cross-polarized (RCP→LCP) channel and superposition states (ℓ = +1 and ℓ = −1) in the copolarized (RCP→RCP) channel. This strategy overcomes limitations of single-layer metasurfaces in varifocal OAM manipulation, while eliminating the need for active materials or complex control systems.

## 2 Design and principles

### 2.1 Metasurface implementation

**Figure 1:**
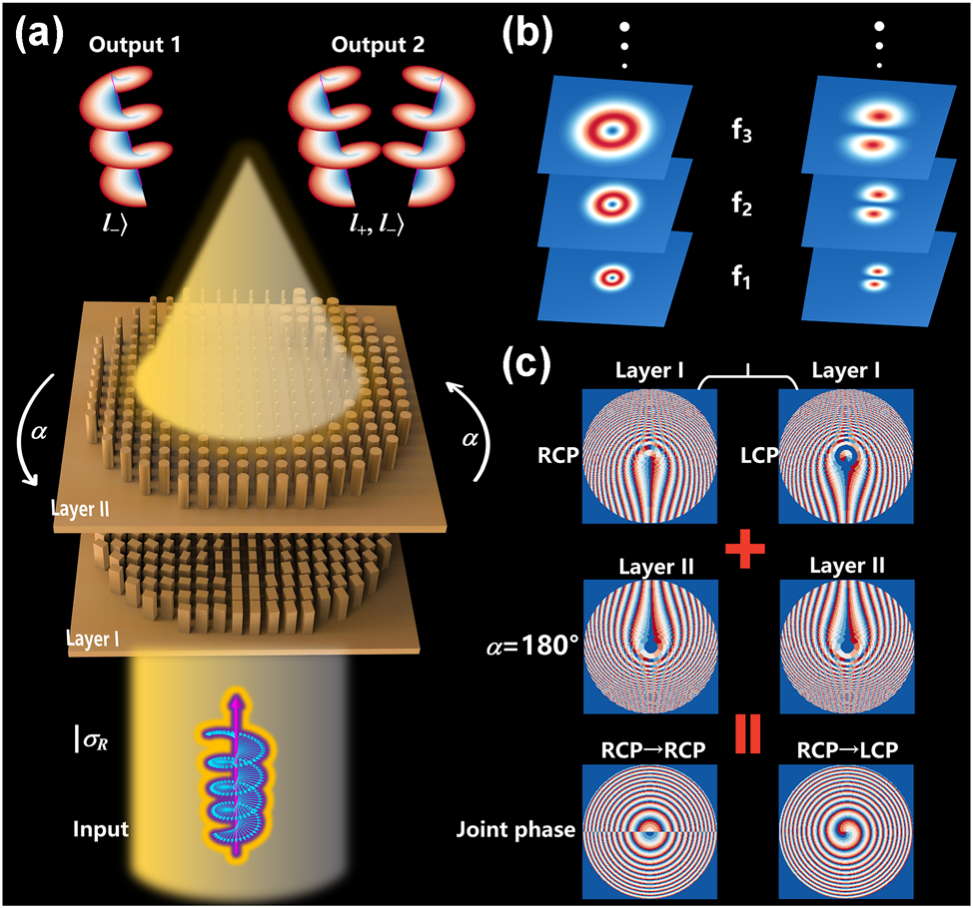
Design illustration of the proposed meta-device. (a) Under RCP illumination, the transmitted orthogonal CP channels generate a focused vortex beam with topological charge ℓ = −1 and a superposition state of vortices with topological charges ℓ = −1 and ℓ = +1, respectively. (b) The focal length dynamically varies with the rotation angle of Layer II at LCP/RCP channel. (c) Phase distribution of Layer I, Layer II at a rotation angle of 180°, and their joint phase profile.

Currently, the reported work on cascaded metasurfaces usually concentrates exclusively on the zoom function and does not involve the polarization dimension, thus limiting the scope of their applications. To increase the versatility of the meta-device, we integrate the traditional cascaded metasurfaces (zoom by rotation) with polarization multiplexing technology, which can expand the multidimensional optical field modulation through the polarization dimension while maintaining the dynamic zoom capability, and catalyze innovative applications. A schematic representation of the designed cascaded metasurface is showcased in Figure 1a, which comprises two layers of strictly aligned coaxial metasurfaces (Layer I and Layer II). Wherein, Layer I is composed of rectangular pillars with polarization conversion properties, and Layer II is made up of cylinders with polarization maintaining properties. At incidence of RCP, a focused vortex beam with topological charge ℓ = −1 can be generated in the LCP channel at the predefined focal plane of the transmission end, while it exhibits a vortex superposition state of ℓ = +1 and ℓ = −1 in the RCP channel. Simultaneously, the focal length of the focused beam is continually varied by rotating the Layer II. The reason that it is possible to produce different types of OAM focused beams in the orthogonal circularly polarized (CP) channel is that an independent phase encoding of the orthogonal CP channel is used for Layer I. Thus, the phase distribution of Layer I can be expressed as [38], [39],

(1) $$\phi_{1L}\left(r,\alpha_{0}\right)=round\left(\frac{r^{2}}{\lambda f}\right)\alpha_{0}+\ell_{1}\zeta$$

(2) $$\phi_{1R}\left(r,\alpha_{0}\right)=round\left(\frac{r^{2}}{\lambda f}\right)\alpha_{0}+\ell_{2}\zeta$$

Here, the round (…) function denotes rounding the argument to the nearest integer, ensuring that the phase shift varies by integer multiples of 2π to prevent aliasing artifacts, Where r and *α*_0_ are the polar coordinate parameters [40], *λ* is operating wavelength, *f* represents the predefined focal length, the parameters *ℓ*_1_ and *ℓ*_2_ represent the topological charges assigned to the RCP→LCP and LCP→RCP channels. Here, we set *ℓ*_*1*_ = −1, *ℓ*_2_ = +1, so that a focused vortex beam with topological charge *ℓ*_*1*_ = −1 can be detected in the transmitted field within the LCP channel when the incidence is RCP, while the topological charge *ℓ*_*1*_ = −1, *ℓ*_2_ = +1 superposition state is monitored in the RCP channel. The variable $\zeta =\arctan\left(y/x\right)$ denotes the polar angle used to encode the vortex phase. The mechanism for realizing the varifocal is the rotations of Layer II relatively to Layer I, which can be expressed as follows,

(3) $$\phi_{2}\left(r,\alpha_{0}\right)=-round\left(\frac{r^{2}}{\lambda f}\right)\alpha_{0}$$

When Layer II is rotated by an angle *α*, the phase of Layer II is changed to,

(4) $$\phi_{2}\left(r,\alpha_{0};\alpha \right)=-round\left(\frac{r^{2}}{\lambda f}\right)\left(\alpha_{0}-\alpha \right)$$

Therefore, the distribution resulting from the combination of Layer I and Layer II can be changed as the rotation of Layer II. According to Eqs. (1)–(4), the relationship between the focal length *F* and the rotation angle *α* can be derived as [41],

(5) $$F=\frac{\pi }{C\lambda \alpha }$$

where *C* is a constant that can be expressed as, $C=\frac{1}{\lambda f}$ . Therefore, the numerical aperture (NA) can be represented as,

(6) $$NA=\sin\left(\tan^{-1}\frac{L}{2\cdot F}\right)$$

Here, *L* denotes the diameter of the metasurface, as governed by Equations (5) and (6), when the incident wavelength is fixed and the initial focal length is prescribed, the focal length undergoes continuous modulation with the angular displacement α (as illustrated in Figure 1b), thereby inducing a corresponding variation in the numerical aperture of the system. To elucidate the advanced manipulation of polarization degree of freedom, Figure 1c presents a representative case with rotation angle α = 180°. The systematic demonstration is the implementation of independent phase encoding for orthogonal CP channels in Layer I. It is then subsequently revealed how their coherent combination with the phase profile of Layer II enables precise control over the joint phase distributions corresponding to distinct polarization channels. It shows that the phase of Layer I exhibits a distinct phase distribution in the LCP channel and the RCP channel, and by combining the phase with that of Layer II, the joint phases in different polarization channels also show significant differences.

### 2.2 Meta-atoms design

**Figure 2:**
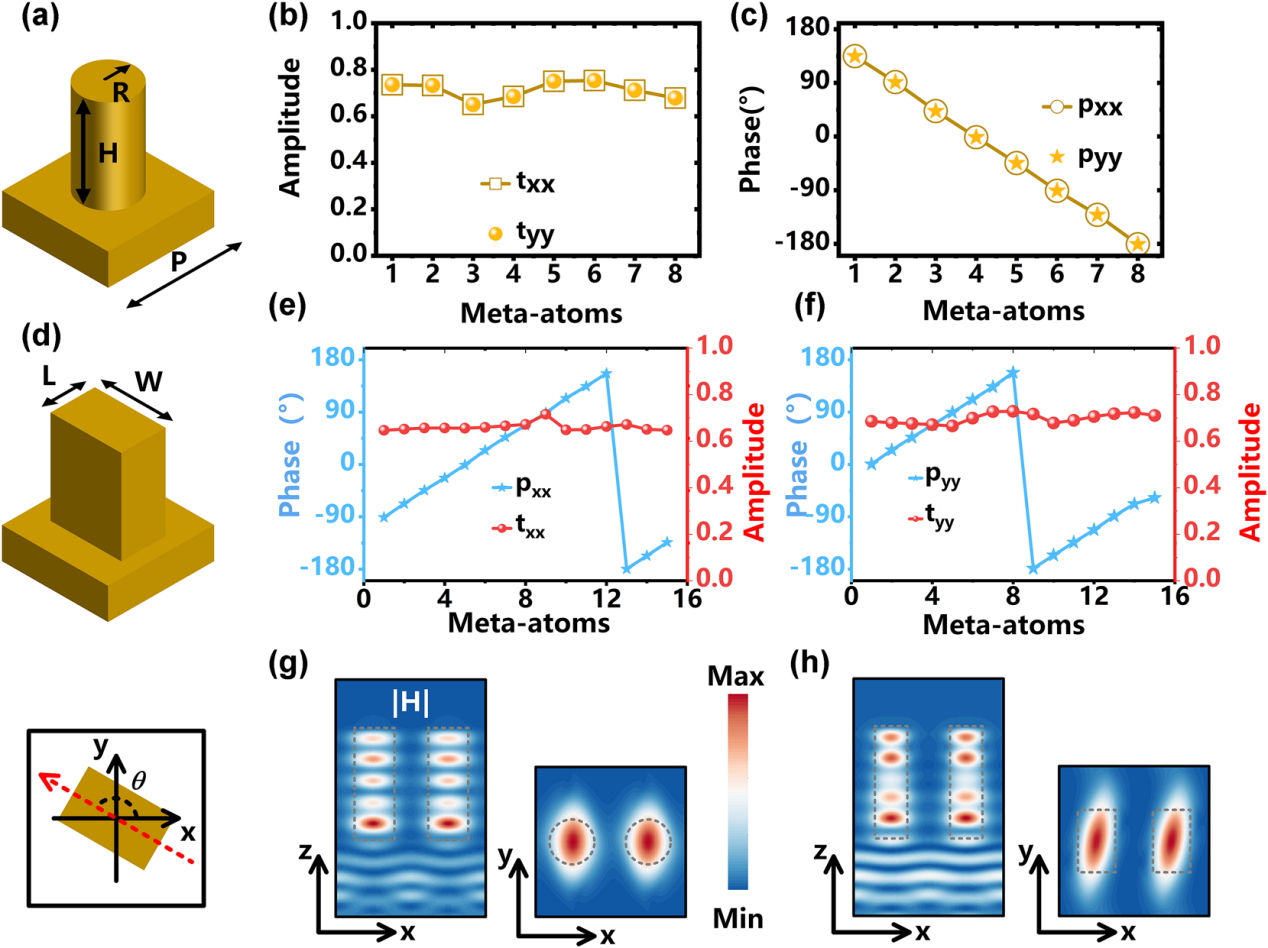
Transmission characteristics of selected meta-atoms for assembling the meta-device. (a) Meta-atoms selected for constructing Layer II with polarization-maintaining properties. (b) Transmission amplitude and (c) phase delay of cylindrical dielectric pillars (labeled 1 to 8) achieving full 2π phase coverage. (d) Side and top views of meta-atoms used for Layer I with polarization conversion functionality. (e) Under x-LP incidence, phase response and transmission amplitude of rectangular dielectric pillars (labeled 1 to 15) achieving 2π propagation phase coverage. (f) Under y-LP incidence, phase delay and transmission amplitude of rectangular dielectric pillars (labeled 1 to 15) achieving 2π propagation phase coverage. Normalized magnetic field distributions of (g) cylindrical dielectric pillar under periodic boundary conditions and (h) rectangular dielectric pillar under periodic boundary conditions.

All meta-atoms constituting the metasurface were fabricated from high-resistivity silicon (*ε* = 11.9) with a fixed lattice period P = 150 μm and pillar height H = 200 μm. For Layer II, which operates independently of polarization control, polarization-insensitive cylindrical pillars were adopted due to their structural symmetry (Figure 2a). To design Layer II, a parametric sweep of the pillar radius R was conducted using CST Microwave Studio, systematically evaluating phase shifts and transmission amplitudes under both x- and y-LP illumination. The optimization process prioritized two objectives: achieving full 0–2π phase coverage and maintaining high and uniform transmission amplitudes. Eight cylindrical meta-atoms were selected based on these criteria, as their polarization-independent properities ensured identical phase retardation and transmission responses for both x-LP and y-LP incidences (Figure 2b). This configuration gradient from −π to 7π/8, meaning that the phase of the 8 meta-atoms successfully covers the target 0-2π phase range while preserving amplitude within a range of 0.6–0.8, thereby ensuring excellent manipulation of the metasurface. As for the design of the meta-atoms in Layer I, a spin-decoupling strategy is employed by leveraging the superposition of propagation phase and geometric phase, which is expressed as [42], [43], [44], [45],

(7) $$\left\{\begin{array}{c} \varphi_{xx}=\left(\varphi_{\mathrm{LCP}}+\varphi_{\mathrm{RCP}}\right)/2\;\\ \varphi_{yy}=\left(\varphi_{\mathrm{LCP}}+\varphi_{\mathrm{RCP}}\right)/2-\pi /2\;\\ \theta =\left(\varphi_{\mathrm{RCP}}-\varphi_{\mathrm{LCP}}\right)/4\; \end{array}\right.$$

Where *φ*_*xx*_ and *φ*_*yy*_ are the propagation phase obtained under orthogonal linear polarization incidence (*x* and *y* directions), *φ*_*LCP*_ and *φ*_*RCP*_ denote the phase modulation of the meta-atoms for LCP and RCP incidence, respectively, *θ* is the rotation angle of the meta-atom, which directly modulates the geometric phase.

For the design of Layer I, anisotropic rectangular pillars with polarization conversion capabilities were selected as meta-atoms. By implementing a spin-decoupling strategy that combines propagation phase and geometric phase modulation, this approach enables independent control over both LCP/RCP components, thereby achieving fully decoupled manipulation of orthogonal polarization channels. To satisfy the requirements of Eq. (7), a dual-parameter sweep of the rectangular pillar dimensions (length L and width W) was performed, the scanning range for the parameters is set between 30 and 140 μm, with a step size of 1 μm. From the parameterized simulation data, 15 meta-atoms were selected based on their transmission characteristics (more details in Section S1, Supporting Information). As shown in Figure 2e and f, the 15-unit phase gradient from −π to 7π/8 achieves full 2π coverage based on the principle of principle of spin-decoupling under both x-and y-LP incidences, with transmission amplitudes consistently maintained within the range of 0.6–0.8. Notably, the phase difference between orthogonal polarizations is fixed at π/2, characteristic of a quarter-wave plate. To gain fundamental insights into the electromagnetic responses, we systematically investigated the normalized magnetic field distributions of meta-atoms under periodic boundary conditions. As demonstrated in Figure 2g and h, the incident wave energy exhibits strong confinement within the high-permittivity dielectric pillars, indicating effective electromagnetic field localization. This confinement effect suggests negligible interlayer coupling within the metasurface architecture, which preserves the operational independence of individual functional layers. The observed energy localization mechanism inherently enhances design robustness by minimizing cross-layer interference, thereby ensuring reliable performance under varying illumination conditions.

## 3 Results and discussion

**Figure 3:**
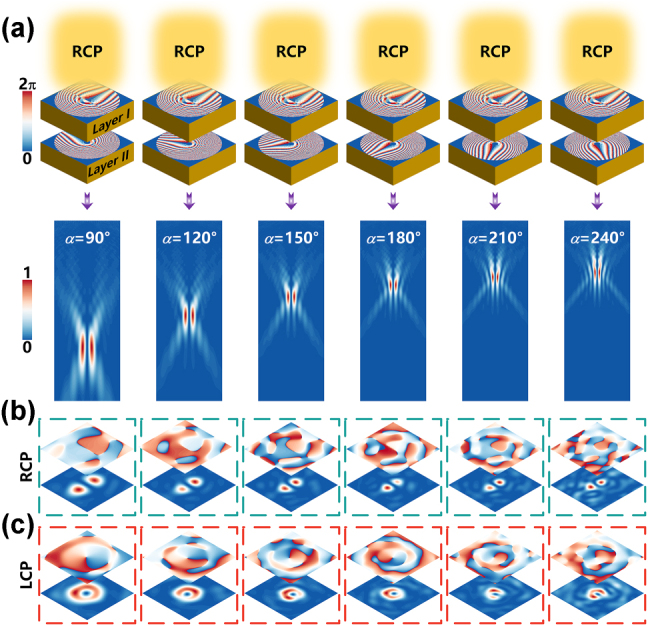
Simulated results of the metasurface device under RCP illumination. (a) Phase distribution of Layer I and Layer II at varying relative rotation angles α and corresponding cross-sectional electric field profiles in the x-z plane as α increases progressively from 90° to 240°. Orthogonal CP channel responses in the x-y plane: (b) electric field intensity and phase distribution in the RCP channel, (c) corresponding profiles in the LCP channel.

In order to confirm the feasibility of the designed strategy, we have carried out an analog simulation in CST, as shown in Figure 3. Figure 3 comprehensively demonstrates the reconfigurable performance of the device under RCP illumination, highlighting its dynamic focal-length modulation and polarization-selective functionalities. The phase distributions of Layer I and Layer II (rotationally tunable with angle *α*) are shown in Figure 3a. The rotational dynamics of Layer II’s phase profile about its central axis, observed along the wave incidence direction, enables precise focal length modulation through counterclockwise angular displacement (*α*). As α increases from 90° to 240°, systematic analysis of the transmitted electric field distributions demonstrates a progressive focal shift along the negative *z*-axis, accompanied by continuous focal length reduction from 26.6λ to 9.9λ. Specifically, parametric analysis reveals angle-dependent focal tuning characterized by discrete transitions: 90° (26.6λ), 120° (21.0λ), 150° (16.6λ), 180° (14λ), 210° (11.4λ), and 240° (9.9λ), the operating wavelength *λ* = 333 μm (at 0.9 THz). The quantified α-f relationship confirms rotational phase engineering as an effective mechanism for achieving submillimeter-precision focal control. Polarization-resolved analysis in the x-y plane provides deeper insights into the metasurface’s spin-decoupling capability. In the RCP channel (Figure 3b), the intensity profile exhibits a dual-lobe distribution with a continuous dark line separating the lobes. The corresponding phase profile displays an abrupt π-phase jump along the nodal line, indicative of the coaxial superposition of ℓ = +1 and ℓ = −1 vortex beams. Conversely, the LCP channel (Figure 3c) demonstrates an annular intensity pattern containing a central dark core (phase singularity), accompanied by a helical phase distribution maintaining the original topological charge of ℓ = −1. The polarization-multiplexed symmetry of the device is further validated under left-handed circularly polarized (LCP) illumination (as detailed in Supporting Information S2). Numerical simulations reveal that the LCP→RCP channel generates a vortex beam with ℓ = +1, while the LCP→LCP channel exhibits a superimposed state ℓ = +1/−1, mirroring the inverse symmetry of the RCP incidence results. This reciprocity underscores the robustness of the spin-decoupled phase modulation mechanism across orthogonal polarization channels. These collective findings establish that rotation-tuned cascaded metasurfaces enable dynamic manipulation of both focal properties and polarization states.

**Figure 4:**
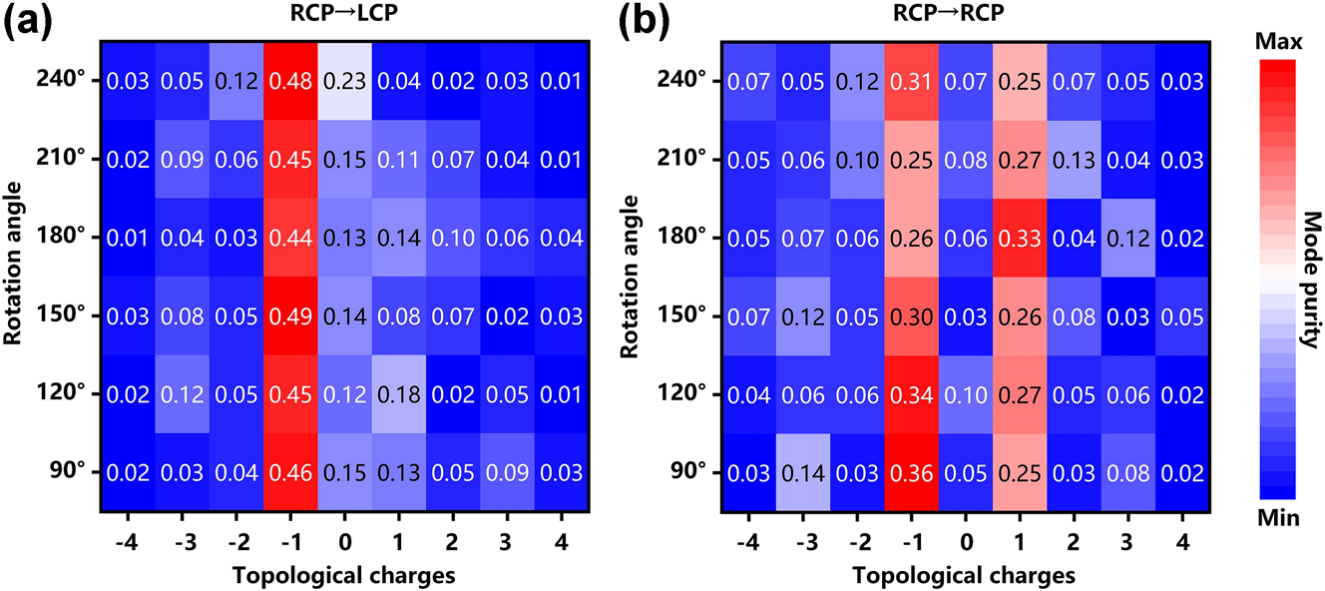
Mode purity analysis of simulation results obtained from RCP incidence. The calculated results corresponding to different rotation angles obtained within (a) RCP→LCP channel and (b) RCP→RCP channel, respectively.

To validate the fidelity of the simulated electric field distributions, the pure ℓ = −1 vortex mode and the superimposed ℓ = +1/−1 hybrid states – a comprehensive modal purity analysis was conducted on the numerically obtained field profiles across orthogonal polarization channels under RCP illumination, as depicted in Figure 4. The mode purity was quantified through spiral harmonic decomposition, wherein the simulated electric field profiles E(r, ϕ) at the focal plane were projected onto a basis of orthogonal Laguerre–Gaussian (LG) modes [46]. The spin-decoupled phase encoding ensures high selectivity for orthogonal polarization channels. It can be seen in Figure 4a, the predominant ℓ = −1 purity in the LCP channel corresponding to the rotation angles from 90° to 240° confirms the efficacy of Moiré phase engineering in suppressing undesired modes. Conversely, Figure 4b demonstrates the OAM mode distribution in the RCP channel, where the superimposed ℓ = +1 and ℓ = −1 states exhibit balanced contributions, with their purity ratios remaining nearly equivalent throughout the angular tuning range, further validating the design’s capability to achieve deterministic polarization-OAM multiplexing. Consequently, ℓ = +1 and ℓ = −1 purities predominantly characterize the distribution of OAM modes within the RCP channel. This quantitative evaluation ensures the reliability of the spin-decoupled phase modulation mechanism and clarifies the distinct OAM characteristics encoded in each polarization channel.

**Figure 5:**
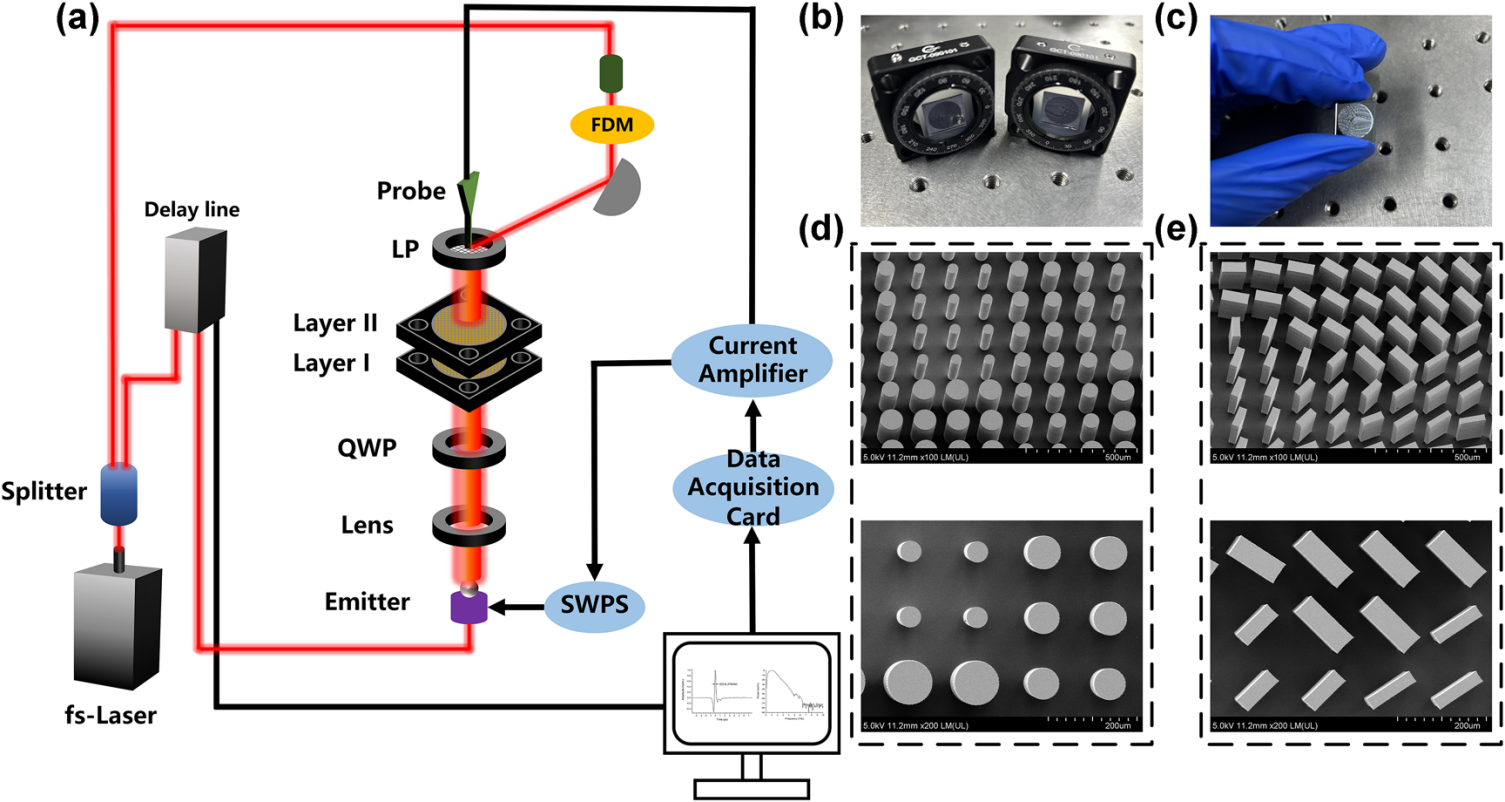
Characterization of THz near-field spectroscopy system and fabricated cascaded metasurfaces. (a) Schematic illustration of the THz time-domain near-field scanning spectroscopy system. (b) The two GCT-090101 fine adjustable polarizer holders embedded in metasurface sample Layer I and Layer II, respectively. (c) Optical photograph of the metasurface sample. (d–e) Magnified scanning electron microscopy (SEM) images of (d) Layer II and (e) Layer I.

To experimentally validate the proposed methodology, we fabricated the metasurface samples of Layer I and Layer II and conducted systematic characterization using the measurement platform. As shown in Figure 5, the experimental configuration and structural analysis collectively demonstrate the feasibility of our approach. Figure 5a depicts the customized THz near-field scanning spectroscopy system for characterizing the focusing behavior of the metasurface device. The system employs a precision microprobe to record both amplitude and phase profiles across the focal plane under linearly polarized (LP) illumination, pixel by pixel at an operating wavelength *λ* = 333 μm. Fabricated Layer I and Layer II samples are mounted in coaxially aligned GCT-090101 fine adjustable polarizer holders (Daheng Optics, China), ensuring mechanical stability during scanning [47]. Device fabrication combines ultraviolet (UV) photolithography and inductively coupled plasma (ICP) etching on 500-μm-thick high-resistivity silicon substrates, chosen to balance optical efficiency with minimal THz absorption [48], [49]. Fabricated samples of Layer I and Layer II were securely mounted in coaxially aligned two GCT-090101 holders (Figure 5b). This system achieves submillimeter alignment precision between the two metasurfaces through four 6-mm through-holes, enabling direct coaxial integration with mounting rods. A rotational stage provides 360° angular displacement with 2° resolution, maintaining repeatable positioning through 30° step intervals, ensuring mechanical stability and angular reproducibility during optical testing. Figure 5c presents an image of the fabricated metasurface, measuring 1.4 × 1.4  cm^2^ overall with an effective area of 0.36π cm^2^. The structure comprises an 80 × 80 array of meta-atoms with a 150 μm periodicity. The nanoscale structural details can be revealed through high-resolution SEM images (Figure 5d and e). Layer II (Figure 5d) demonstrates uniformly patterned cylindrical silicon pillars with sharp sidewall profiles and submicron surface roughness. In contrast, Layer I (Figure 5e) exhibits rectangular pillars whose geometrically distinct edges maintain comparable fabrication quality. Both structures confirm exceptional process control through their defect-free periodic arrangements and surface uniformity. These nanoscale features, combined with the measured angular resolution (2°) and alignment precision (submillimeter scale) of the rotational stage, collectively validate the experimental component’s capability to probe zoom phenomena.

**Figure 6:**
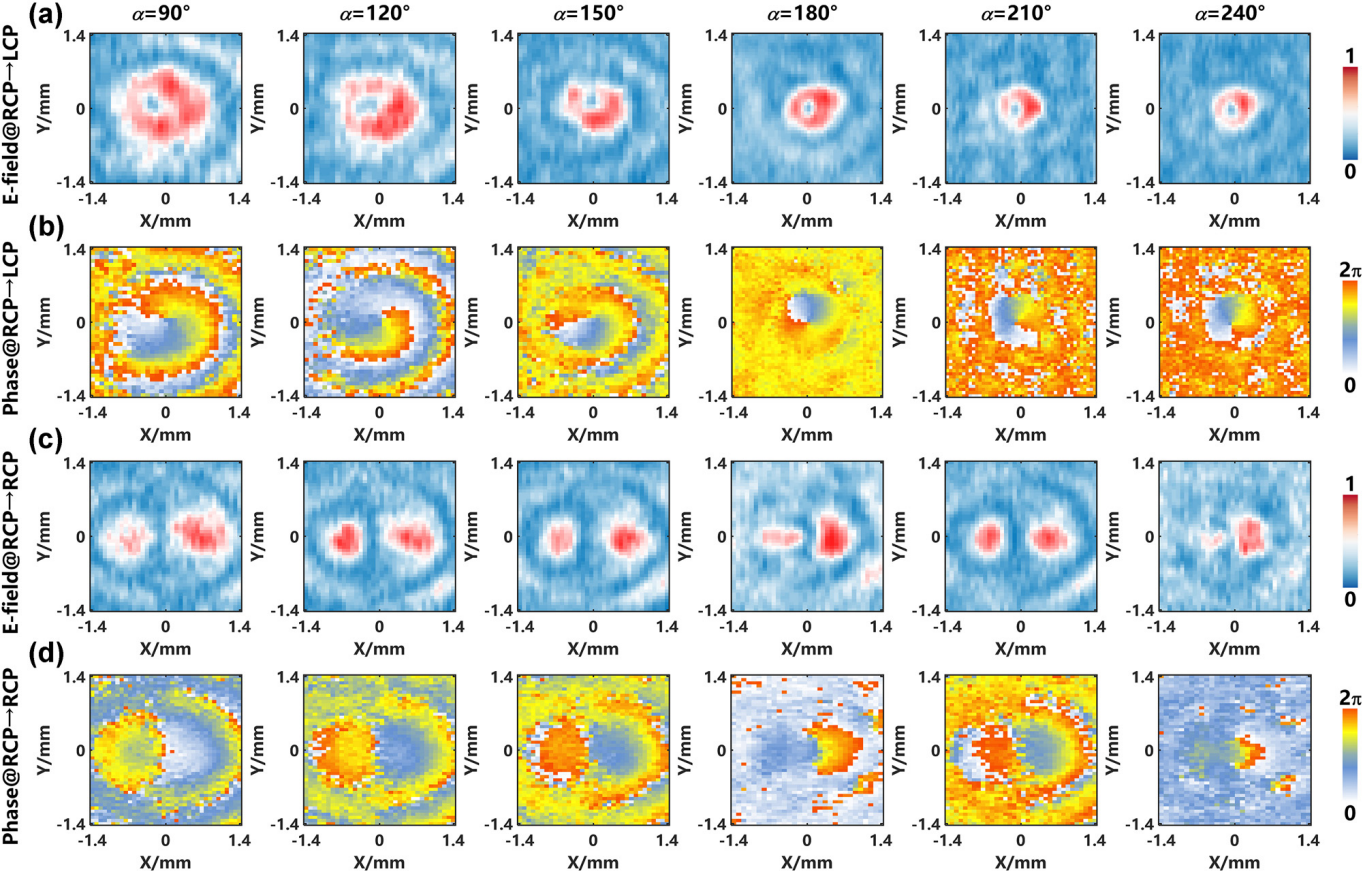
Experimental result. (a) Normalized intensity profiles and (b) corresponding phase distributions of the RCP to LCP conversion channel, generating a pure vortex beam with topological charge ℓ = −1. Measurements span rotation angles from 90° to 240° in 30° increments. (c) Normalized intensity profiles and (d) phase distributions of the RCP to RCP channel, producing a superposition state of ℓ = −1 and ℓ = +1 topological charges.

The rotational tuning precision of our dual-layer metasurface platform, enabled by the submillimeter alignment mechanism and angular control system detailed in Figure 5, achieves polarization-selective vortex generation with programmable orbital angular momentum. In the RCP-to-LCP conversion channel, a vortex beam with topological charge ℓ = −1 is generated with the focal length decreasing over a range of rotation angles from 90° to 240° (in steps of 30°). Figure 6a and b systematically document this behavior, showing how each 30° rotation distributes the focal plane’s intensity into concentric annular profiles while maintaining a spiral phase front with a single 2π-periodic singularity. Conversely, the RCP-to-RCP channel exploits interference effects to create a hybrid state combining ℓ = −1 and ℓ = +1 topological charges, as evidenced in Figure 6c and d. Angular rotation induces progressive modifications in both intensity and phase domains: the intensity profile transitions from a central null to two lobes (Figure 6c), while the phase maps (Figure 6d) exhibit distinct discontinuities. Both channels collectively demonstrate the platform’s dual operational paradigm – generating either deterministic single-charge vortices or reconfigurable hybrid states through the same physical architecture, governed solely by input polarization and rotational alignment.

**Figure 7:**
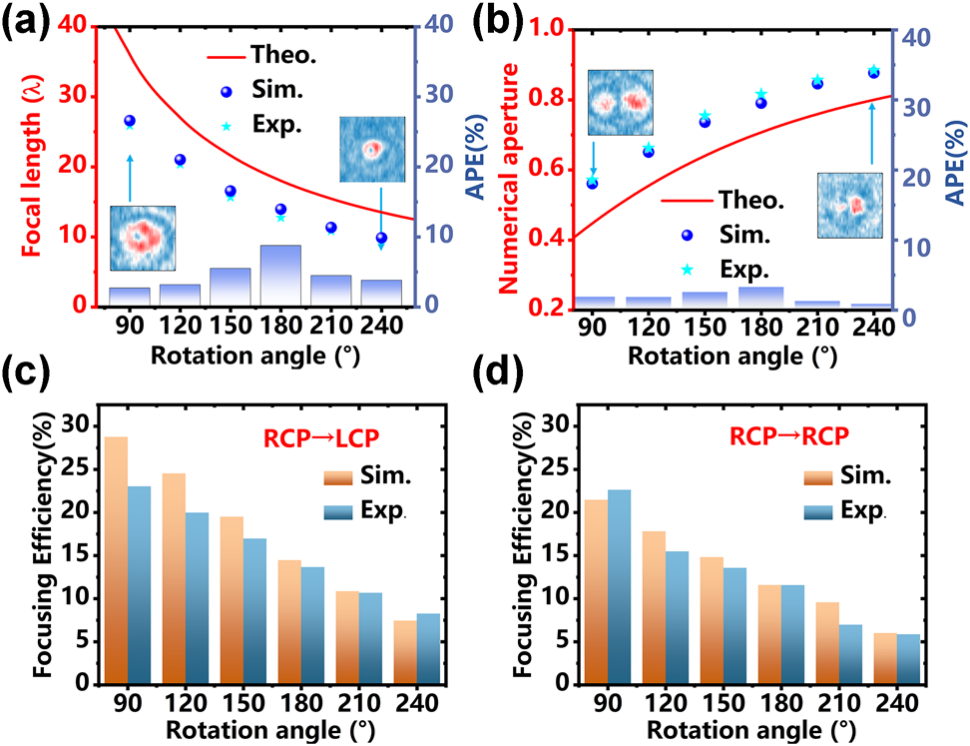
The device’s performance characterization under rotational variations. (a) Measured focal lengths versus rotation angle (90°–240°) with comparisons to theoretical predictions and simulations. Solid line represents theoretical values, circle markers denote simulated results, and star markers indicate experimental data. The purple column shows the absolute percentage error (APE) between simulation data and experimental data. (b) Numerical aperture (NA) evolution across the same angular range, showing agreement between analytical calculations (solid line), electromagnetic simulations (spherical markers), and measurements (star markers). The purple column illustrates the APE between the simulation data and the experimental data. Polarization-resolved focusing efficiencies for both (c) cross-polarization (RCP-to-LCP) conversion and (d) copolarization (RCP-to-RCP transmission).

The angular-dependent characteristics of the metasurface were systematically investigated through rotational variations in 30° increments from 90° to 240°. As illustrated in Figure 7a, the focal length demonstrates a monotonic decrease with increasing rotation angle, exhibiting excellent agreement between electromagnetic simulations (spherical marker) and experimental measurements (star marker). Specifically, the simulated focal length reduces from 26.6λ to 9.9λ (62.8 % reduction), while experimental results show a corresponding decrease from 25.9λ to 9.5λ (63.3% reduction). The absolute percent error (APE) between simulation and experiment remains below 8 % across all angles, peaking at 180° rotation. Figure 7b reveals a complementary relationship between numerical aperture (NA) and rotation angle, where NA increases from 0.57 to 0.89 as the Layer II rotates from 90° to 240°. This inverse correlation with focal length evolution follows the fundamental relationship as depicted in Eq. (6). The experimental measurements (star marker) show remarkable consistency with simulations (spherical marker), achieving an APE below 4 %. The angularly programmable NA adjustment confirms the metasurface’s capability for tunable wave confinement. Figure 7c systematically quantifies the angular dependence of focusing efficiency across cross-polarized (RCP-to-LCP) channel, which can be defined as: $\eta_{f}={\tilde{E}}_{f}/{\tilde{E}}_{\text{sum}}=\int \int E_{f}\left(E_{f}\geq \left(E_{\max}/e\right)\right)/\int \int E_{f}$ , where ${\tilde{E}}_{f}$ is the integral of the focused field distribution over a range greater than *E*_max_/*e*, and ${\tilde{E}}_{\text{sum}}$ represents the integral of the field distribution at the entire focal plane [50]. The cross-polarization focusing efficiency demonstrates a pronounced angular sensitivity, decreasing from 28.7 % to 7.44 % in simulations (23.0 %–8.25 % experimentally) as rotation progresses from 90° to 240°. This 74.1 % relative reduction (simulated) and 64.1 % experimental decrease primarily originate from progressive phase mismatch. Notably, as seen in Figure 7d, the copolarized channel (RCP-to-RCP) exhibits analogous angular attenuation. Simulated RCP-to-RCP focusing efficiency decreases from 21.5 % to 5.9 % (72.6 % reduction), while experimental results follow this trend with 22.6 %–5.9 % reduction (73.9 %), maintaining <5 % deviation from simulations. This parallelism stems from shared angular-modulated scattering losses. Both polarization channels maintain >5 % efficiency across all angles, confirming robust operation for practical polarization-multiplexed systems.

## 4 Conclusions

In summary, this work presents a cascaded dielectric metasurface platform that achieves synergistic control of polarization-multiplexed wavefronts and dynamically tunable focusing, addressing critical limitations in conventional single-layer metasurfaces constrained by static responses and restricted polarization-channel independence. The proposed dual-layer device leverages distinct polarization-phase coupling mechanisms to enable multifunctional wave manipulation. In Layer I, anisotropic rectangular meta-atoms with polarization-sensitive birefringence are strategically designed to implement spin-decoupled phase encoding. This allows independent wavefront tailoring for orthogonal CP channels. Layer II, composed of rotationally symmetric cylindrical meta-atoms, introduces a polarization-insensitive phase. Notably, under RCP illumination, the device simultaneously generates two distinct vortex states: an LCP focus vortex beam with topological charge ℓ = −1 and a copolarized RCP vortex beam exhibiting a superposition of ℓ = +1 and −1 topological charges.

The coaxial integration of these layers within two mechanically tunable GCT-090101 holders establishes a dynamic wave-field manipulation platform, enabling continuous focal length tuning without altering the metasurface geometry or material composition. Experimental validation demonstrates that a rotation angle sweep from 90° to 240° adjusts the focal length from 25.9λ to 9.5λ, achieving a 63.3 % tuning range while maintaining good focusing efficiency. Unlike previous tunable metasurfaces relying on active materials or complex reconfiguration mechanisms, our approach achieves simultaneous polarization encoding, orbital angular momentum multiplexing, and real-time focal adjustment. Such capabilities hold transformative potential for applications ranging from miniaturized LiDAR systems requiring rapid depth scanning to compact quantum optics platforms demanding polarization-entangled photon manipulation.

## Supplementary Material

Supplementary Material Details
