# Supplementary material for: Cascaded metasurface for polarization-dependent varifocal vortex beam manipulation
Source: Nanophotonics. 2025 Jul 9;14(17):2881–90. doi: 10.1515/nanoph-2025-0153 (PMC12397744; doi:10.1515/nanoph-2025-0153)
Supplement: Supplementary file 1 — Supplementary Material Details [file j_nanoph-2025-0153_suppl_001.docx]

**Supporting Information**

**Cascaded metasurface for polarization-dependent varifocal vortex beam manipulation**

**Wenhui Xu, Chenghui Zhao, Hui Li, Jie Li, Qi Tan, Yufei Liu, Hang Xu, Yun Shen, and Jianquan Yao**

**S1. Geometric parameters of the 15 rectangular meta-atoms.**

To identify spin-decoupled meta-atoms for Layer I, a dual-parameter sweep of the rectangular pillar dimensions (length L and width W) was systematically performed in CST Microwave Studio, spanning a geometric range of 30-140 μm with a step resolution of 1 μm. This exhaustive parametric exploration prioritized dual-objective optimization: achieving full 0-2π phase coverage and maintaining uniform transmission amplitudes. From the simulated dataset, 15 meta-atoms meeting the spin-decoupling criteria were selected, as detailed by their geometric parameters in Table S1. The chosen units exhibit a phase gradient from −π to 7π/8 under x- and y-polarized incidences, fulfilling the requirements for independent polarization-phase control.

**Table S1**. Geometric parameters of the 15 rectangular meta-atoms.

|  | 1 | 2 | 3 | 4 | 5 | 6 | 7 | 8 | 9 | 10 | 11 | 12 | 13 | 14 | 15 |
| --- | --- | --- | --- | --- | --- | --- | --- | --- | --- | --- | --- | --- | --- | --- | --- |
| L(μm) | 63 | 62 | 61 | 59 | 56 | 51 | 47 | 39 | 36 | 88 | 77 | 71 | 67 | 65 | 65 |
| W(μm) | 87 | 82 | 78 | 75 | 74 | 74 | 74 | 77 | 35 | 113 | 135 | 133 | 129 | 121 | 104 |

**S2. The transmitted electric fields and phase distributions of different channels obtained under LCP incidence.**

To further investigate the operational symmetry of the device, numerical simulations were conducted in CST Microwave Studio under LCP illumination. The resulting electric field and phase distributions in transmission mode are presented in Fig. S2. As shown, the cross-polarized (LCP→RCP) channel generates a vortex beam carrying a topological charge of ℓ = +1, while the co-polarized (LCP→LCP) channel exhibits a hybrid state comprising superimposed ℓ = +1 and ℓ = −1 vortices at different rotation angles (90°-240°). These observations are inversely symmetric to those obtained under RCP illumination, as demonstrated in the main text. This reciprocal behavior originates from the spin-decoupled phase modulation mechanism inherent in the metasurface design. Such symmetry confirms the robust polarization-multiplexed functionality of the cascaded platform for deterministic OAM state generation.


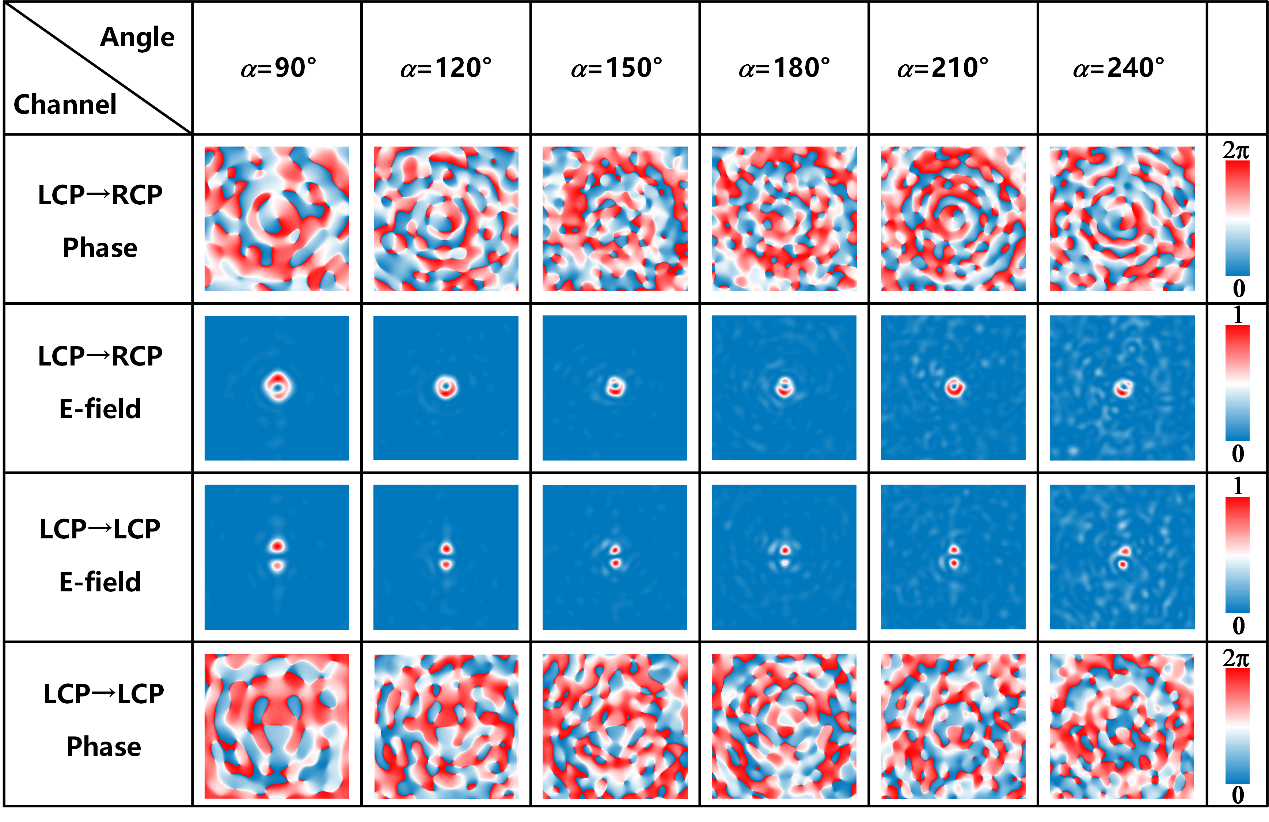


**Fig. S2:** The transmitted electric fields and phase distributions of LCP→RCP and LCP→LCP channels obtained under LCP incidence.
